# Unravelling the Gastroprotective Potential of Kefir: Exploring Antioxidant Effects in Preventing Gastric Ulcers

**DOI:** 10.3390/cells12242799

**Published:** 2023-12-08

**Authors:** Larissa Zambom Côco, Rafaela Aires, Glaucimeire Rocha Carvalho, Eduarda de Souza Belisário, Michelle Khai Khun Yap, Fernanda Gobbi Amorim, Javier Conde-Aranda, Breno Valentim Nogueira, Elisardo Corral Vasquez, Thiago de Melo Costa Pereira, Bianca Prandi Campagnaro

**Affiliations:** 1 Laboratory of Translational Physiology and Pharmacology, Pharmaceutical Sciences Graduate Program, Vila Velha University (UVV), Vila Velha 29102-920, ES, Brazil; larissa.coco@uvvnet.com.br (L.Z.C.); rafaela.aires@yahoo.com (R.A.); glaucimeire.rcarvalho@uvvnet.com.br (G.R.C.); eduarda.belisario@uvvnet.com (E.d.S.B.); elisardo.vasquez@uvv.br (E.C.V.); thiago.pereira@uvv.br (T.d.M.C.P.); 2 School of Science, Monash University Malaysia, Bandar Sunway 47500, Selangor, Malaysia; yap.michelle@monash.edu; 3 Laboratory of Mass Spectrometry, Department of Chemistry, University of Liège, 4000 Liège, Belgium; fernandagamorim@gmail.com; 4 Molecular and Cellular Gastroenterology, Health Research Institute of Santiago de Compostela (IDIS), 15706 Santiago de Compostela, Spain; jcondearanda@idisantiago.es; 5 Department of Morphology, Health Sciences Center, Federal University of Espírito Santo (UFES), Vitoria 29047-105, ES, Brazil; breno.nogueira@ufes.br

**Keywords:** gastroprotection, bioprospection, oxidative stress, anti-inflammatory

## Abstract

The present study was conducted to evaluate the protective effect of milk kefir against NSAID-induced gastric ulcers. Male Swiss mice were divided into three groups: control (Vehicle; UHT milk at a dose of 0.3 mL/100 g), proton pump inhibitor (PPI; lansoprazole 30 mg/kg), and 4% milk kefir (Kefir; 0.3 mL/100 g). After 14 days of treatment, gastric ulcer was induced by oral administration of indomethacin (40 mg/kg). Reactive oxygen species (ROS), nitric oxide (NO), DNA content, cellular apoptosis, IL-10 and TNF-α levels, and myeloperoxidase (MPO) enzyme activity were determined. The interaction networks between NADPH oxidase 2 and kefir peptides 1–35 were determined using the Residue Interaction Network Generator (RING) webserver. Pretreatment with kefir for 14 days prevented gastric lesions. In addition, kefir administration reduced ROS production, DNA fragmentation, apoptosis, and TNF-α systemic levels. Simultaneously, kefir increased NO bioavailability in gastric cells and IL-10 systemic levels. A total of 35 kefir peptides showed affinity with NADPH oxidase 2. These findings suggest that the gastroprotective effect of kefir is due to its antioxidant and anti-inflammatory properties. Kefir could be a promising natural therapy for gastric ulcers, opening new perspectives for future research.

## 1. Introduction

Nonsteroidal anti-inflammatory drugs (NSAIDs) are a major class of over-the-counter medications, renowned for their potent analgesic and antipyretic effects [1]. The extensive use of NSAIDs in pain and fever management, as well as their therapeutic role in inflammatory conditions such as chronic pain, rheumatoid arthritis, and postoperative recovery, is well-established in modern pharmacotherapy [2]. However, long-term use of NSAIDs is associated with significant gastrointestinal injury, specifically leading to the development of gastric lesions [3]. Nonsteroidal anti-inflammatory drugs (NSAIDs) are a major class of over-the-counter medications, renowned for their potent analgesic and antipyretic effects [1]. The extensive use of NSAIDs in pain and fever management, as well as their therapeutic role in inflammatory conditions such as chronic pain, rheumatoid arthritis, and postoperative recovery, is well-established in modern pharmacotherapy [2]. However, long-term use of NSAIDs is associated with significant gastrointestinal injury, specifically leading to the development of gastric lesions [3]. While NSAIDs are an important cause of ulcers, factors such as stress, smoking, alcoholism, frequent and indiscriminate use of medications, infection with *Helicobacter pylori*, heredity, increase in gastric acid secretion, and excessive reactive oxygen formation (ROS), among others, can play different roles in the genesis of the disease, and are therefore considered important risk factors for the development of gastric ulcers [4,5,6,7].

The pharmacological effects of NSAIDs are primarily mediated by their inhibition of cyclooxygenase (COX) enzymes, which catalyze the conversion of arachidonic acid into biologically active compounds, including prostaglandins, prostacyclins, and thromboxanes [8]. Among the COX isoforms, COX-1 is expressed in multiple tissues and plays a critical role in maintaining homeostasis [9]. Prostaglandins, which are mediated by COX-1, are essential for protecting the gastrointestinal tract, particularly with respect to mucus production and epithelial defense inhibiting acid gastric secretion [10,11,12]. 

The toxicity of NSAIDs can cause gastric ulceration by reducing cytoprotective mucus, damaging the epithelium, and disrupting the microvasculature [13]. Additionally, the cytotoxicity of NSAIDs contributes to gastric injury by increasing intracellular reactive oxygen species (ROS) and inflammatory cytokines, which ultimately leads to damage to macromolecules [13,14].

The classic pharmacological approach for the treatment of gastric ulcers involves the inhibition of gastric acid secretion using proton pump inhibitors (PPIs) [15,16]. However, prolonged use of PPIs has been associated with micronutrient absorption deficiencies and adverse reactions, including an elevated risk of infections and the development of kidney, liver, and neurological diseases [17]. 

Within this context, there is a growing body of evidence supporting the use of natural therapeutic interventions for the treatment of gastric ulcers, without the associated adverse effects [13,14,18,19,20]. In recent years, kefir, a fermented milk drink, has gained popularity due to its beneficial properties, particularly its notable impact on the gastrointestinal tract [14,21]. Given that kefir has been a part of human consumption for centuries, it is regarded as safe for human health. 

Kefir, a traditional dairy beverage originating from the Caucasus Mountains and Eastern European regions, is crafted through the direct action of kefir grains on milk [22]. Nowadays, its popularity has been expanded worldwide and it is considered a healthy product with high nutritional value. Kefir grains are a consortium of diverse microorganisms, fostering symbiotic relationships that encompass yeasts (*Kluyveromyces*, *Candida*, *Saccharomyces*, and *Pichia*), lactic acid bacteria (*Lactobacillus*, *Lactococcus*, *Leuconostoc*, and *Streptococcus*), and acetic acid bacteria [23,24,25,26]. These microorganisms adhere to a polysaccharide matrix known as kefiran, comprised of branched chains of glucose and galactose [27,28]. This symbiotic relationship categorizes kefir as a functional food [29], attributing its rich nutritional content of fermentation by-products such as organic acids, volatile aromatic products, vitamins, enzymes, and minerals [30]. The microbial consortium within kefir grains generates metabolites with remarkable health-promoting effects, including anti-hypertensive [24,31,32], anti-atherogenic [33,34], anti-diabetes [35,36], anticancer [37,38], anticolitis [39,40], anti-ulcerogenic [14,18], anti-inflammatory [41,42], and antioxidant properties [14,32,41,42]. Notably, kefir exhibits various benefits for the gastrointestinal tract, influencing the modulation of the intestinal microbiota, mitigating inflammatory bowel disease severity, and preserving gastric epithelium integrity [43,44]. The bioactive compounds present in kefir, produced by microorganisms during beverage fermentation and storage, are attributed to these health benefits, including kefiran, exopolysaccharides, bioactive peptides, and organic acids [45]. Therefore, the aim of this study is to assess the effects of milk kefir on gastric cells using an experimental model of gastric ulcer induced by NSAIDs, a class of anti-inflammatory drugs.

## 2. Materials and Methods

### 2.1. Animals

Ten-week-old Swiss mice (~35 g) were included in this study and provided with a normal chow diet. The mice were individually housed in plastic cages under controlled environmental conditions, maintaining a temperature of 22–23 °C and humidity level of 60%. A 12 h light/dark cycle was established in the animal care facility of Vila Velha University (Brazil). The experimental procedures strictly adhered to the guidelines outlined in the National Institutes of Health Guide for Care and Use of Laboratory Animals (NIH- No. 85-23, revised 1996). Ethical approval for the study was obtained from the Institutional Animal Care and Use Committee at Vila Velha University (protocol #524-2019).

### 2.2. Experimental Design

The randomized mice were assigned to three distinct groups as follows: the Vehicle group (n = 8) was administered whole milk at a dosage of 0.3 mL of whole milk/100 g body weight, with its pH adjusted to 5.0. The PPI group (n = 8) received proton pump inhibitor at a dosage of 30 mg/kg (Lansoprazole UlcerStop^®^-Diffucap Chemobras, Química e Farmacêutica LTDA, Rio de Janeiro, RJ, Brazil ). Lastly, the Kefir group (n = 8) was administered milk kefir at a dosage of 0.3 mL/100 g (4% concentration). The administration of all treatments was conducted through gavage and continued for a duration of 14 days.

### 2.3. Kefir Preparation, Administration, and Microbiological Composition

The preparation of Kefir was conducted at the Translational Physiology and Pharmacology Laboratory at Vila Velha University. Kefir grains were incubated with UHT whole milk 4% (*w*/*v*) and maintained at room temperature for 24 h. Subsequently, this mixture was filtered and refrigerated at approximately 6 °C for 24 h. To prevent alterations in the composition of kefir due to storage, after fermentation, the kefir was aliquoted into microtubes and stored at −20 °C until the time of animal treatment [24]. 

The identification of bacteria and yeasts was carried out using the surface spread technique on four different Agar media (Acetobacter, Nutrient, MRS, and Sabouraud, Sigma-Aldrich, St. Louis, MO, USA) under specific conditions of temperature (25, 30, and 37 °C), atmosphere (aerobiosis and anaerobiosis), and time intervals (24, 48, 96, and 120 h). Following this, Gram staining and examination for colony and cell characteristics, as well as catalase, oxidase, coagulase, and bile-esculin activity, were performed on the bacterial isolates. Upon isolation, the species were confirmed using API galleries (BioMérieux, Craponne, France). The microbiological analysis of random samples from the grains used in this study revealed that the predominant microflora of kefir comprised *Acetobacter* spp., *Lactobacillus* spp., *Enterococcus* spp., *Leuconostoc* spp., and *Candida* spp., in a total microbial count of 7.5 × 10^7^ CFU/mL.

### 2.4. Induction of Gastric Ulcer

According to the method suggested by [14], gastric lesions were performed in all experimental groups after the 14th day of treatment. Firstly, the animals were fasted for 16 h, with free access to water. Subsequently, indomethacin (40 mg/kg, purchase from Fagron-Barsbüttel, Barsbüttel, Germany) was administered orally (by gavage). After 6 h, the animals were killed with an overdose of thiopental anesthetic (150 mg/kg *i.p.*, purchased from Thipentax^®^, Cristália, São Paulo, Brazil).

### 2.5. Analysis of Gastric Juice

Gastric juice was collected with 0.9% saline solution, followed by centrifugation at 50× *g* for 10 min, and the supernatant was removed to measure the stomach pH using a pHmeter [14].

### 2.6. Gross Morphology, Ulcer Score, and Protective Index

Stomach images were captured using a digital camera in a photographic studio (Pop Up Studio). The percentage of gastric ulcer was determined using ImageJ software (Image-J 1.35 d, USA, open-source application available at https://imagej.nih.gov/ij/download.html, (accessed on 20 November 2023)) [14]. The classification of the gastric ulcer was scored based on the following parameters: Level 0: normal mucosa; Level 1: vascular injury; Level 2: one or two injuries; Level 3: severe injuries; Level 4: many severe injuries; Level 5: mucosa full of lesions [46].

The ulcer area (mm^2^) was measured using the ImageJ software (Image-J 1.35 d, USA; public domain). Subsequently, the protective index was calculated using the formula (Ulcer area indomethacin (NSAID)—ulcer area treated)/(Ulcer area indomethacin) × 100 = % prevention [47].

### 2.7. Isolation of Gastric Cells

Gastric cells isolation was performed according to [48]. The stomach was triturated and incubated with 1 mL of digestion solution (0.03% type 1 collagenase and 0.1% proteinase K) at 37 °C for 60 min. The cell extract was filtered through a nylon screen (BD Falcon 70 μm) and centrifuged (1700× *g* for 3 min). The cells were resuspended in storage solution (95% SFB and 5% DMSO) and stored at −80 °C until analysis.

### 2.8. Reactive Oxygen Species Production

The production of reactive oxygen species (ROS) in gastric cells was assessed using different dyes: dihydroetidium (DHE, Sigma-Aldrich, St. Louis, MO, USA), 2′,7′-dichlorofluorescein diacetate (DCFH-DA, Sigma-Aldrich, St. Louis, MO, USA), diaminofluorescein (DAF, Sigma-Aldrich, St. Louis, MO, USA), and hydroxyphenylfluorescein (HPF, Sigma-Aldrich, St. Louis, MO, USA). The cells were incubated in the dark with 160 μmol/L of DHE, 20 μmol/L of DCF, 10 μmol/L of HPF for 30 min and 2 μmol/L of DAF for 180 min at 37 °C. The data were acquired using the FACSCanto II and analyzed using FCS Express software version 6.0 (De Novo, Los Angeles, CA, USA). The median fluorescence intensity (MFI) was expressed in arbitrary units (a.u.) to quantify the levels of ROS production [14].

### 2.9. Cell Viability and Apoptosis

The determination of viability and apoptosis of gastric cells was performed using the FACSCanto II flow cytometer (Becton Dickinson Immunocytometry Systems, San Jose, CA, USA). The cells were suspended in buffer solution and incubated with annexin V and propidium iodide (Apoptosis Detection Kit I, BD Pharmingen, Becton Dickinson, San Diego, CA, USA) for 15 min at 37 °C. The data were collected using the FCS Express software version 6.0 (De Novo, Los Angeles, CA, USA). Cells that tested negative for both markers were categorized as viable, whereas cells positive for annexin V-FITC were classified as apoptotic. The results were presented as cell percentages [49].

### 2.10. DNA Fragmentation

To assess DNA content, gastric cells were washed with PBS and centrifuged at 200× *g* for 10 min. Subsequently, the cells were incubated with staining solution (200 μL of RNAse A (20 mg/mL), 800μL of PI (500 μg/mL), 20μL of Triton X-100, Q.s.p., and 20 mL of PBS1x) for 30 min at 4 °C. Following the incubation, the cells were centrifuged at 200× *g* for 10 min. The presence of fragmented DNA (sub-G0/G1 region) in the cells was analyzed using the FCS Express software version 6.0 (De Novo, Los Angeles, CA, USA) [50].

### 2.11. Plasma Levels of IL-10 and TNF-α

The cytokine levels (IL-10 and TNF-alpha) were determined in the plasma by flow cytometry using a Cytometry Bead Array (CBA—Mouse Inflammation Kit), according to the manufacturer’s instruction (BD Biosciences, San Jose, CA, USA). The analyses were performed using the FACSCanto II (BD Biosciences, San Jose, USA). The samples were quantified by comparison with the standard cytokine curve using FCAP Array software version 3.0 (BD Biosciences, San Jose, CA, USA), and the results were expressed in pg/mL [41].

### 2.12. Myeloperoxidase Activity

The myeloperoxidase (MPO) activity was performed according to [32], with minor modifications. The plasma sample was mixed with o-dianisidine solution, placed in a microplate, and the reading was performed at 460 nm in the time from 0 min to 15 min. The results were expressed as units of MPO per milligram of protein (U/mg^−1^ of protein). The protein content was quantified using the Bradford method [51]. The analyses were conducted using spectrophotometer (Synergyx H1 Hybrid Multidetection, Biotek, Winooski, VT, USA).

### 2.13. Histopathological Evaluation of Gastric Damage

To visualize the gastric ulcer microscopically, the stomach was removed, washed, and immersed in a 4% paraformaldehyde solution for fixation. The sample was then dehydrated, blocked, and cross-sectioned at 5 µm before staining with hematoxylin and eosin (HE). The slides were photographed using an Olympus AX70 optical microscope with a Zeiss camera AxioCam ERc5S image acquisition system (Tokyo, Japan). Mucus production was evaluated by identifying glycoproteins, particularly mucin, using staining slides with periodic acid-Schiff (PAS), the classical and most versatile of the carbohydrate stain that demonstrates neutral mucins. The quantification of mucin in gastric tissue was conducted using ImageJ software (version 1.35d, USA; public domain). The analysis of the percentage of mucin-specific staining intensity was normalized based on the total area of the sample.

For morphometric analysis, the structure, edema in the submucosal layer, and the presence of inflammatory cells in the submucosa were examined. To assess the epithelial erosion score, the criteria established by [52] were followed: Level 0: Without tissue alteration; level 1: Superficial changes in the tissue; level 2: Small changes in tissue structures and cellular changes; level 3: Advanced changes throughout the mesh structure. For the evaluation of edema in the submucosa and the presence of inflammatory cells, the parameters were analyzed according to [20]: Level 0: Negative; level 1: Weak; level 2: Moderate; level 3: Strong.

### 2.14. Gastric Scanning Electron Microscopy

Gastric scanning electron microscopy (SEM) was performed according to the published protocol by [32], with minor modifications. The stomach was removed, washed, and immersed in cold fixing solution (2.5% glutaraldehyde, 2% paraformaldehyde, and 0.1 M cacodylate buffer, pH 7.2). Subsequently, the samples were dehydrated in ethanol and washed at room temperature, using ethanol concentration of 30%, 50%, 70%, 90% for 10 min each, followed by a final wash in 100% ethanol for 30 min. The samples were placed in an aluminum stub (Electron Microscopy Sciences, Washington, DC, USA), and the drying was carried out in CO_2_ (Autosandri-815, Tousimis, Rockville, MD, USA) for 40 min, followed by coating with 10 nm of pure gold in a vacuum sprinkler (Desk V, Denton Vacuum, Moorestown, NJ, USA) for 15 min. Analysis and photography were performed using a scanning electron microscope (Jeol, Akishima, Japan, JEM-6610 LV).

### 2.15. Construction of PDB Structure of NADPH Oxidase 2

Based on [53] publication, 35 peptides from the proteomic analyses of Kefir were selected to be analyzed in the present study. These peptides were then subjected to docking experiments with NADPH oxidase 2 to investigate their potential antioxidant activities.

The protein data bank (PDB) structure of NADPH oxidase 2 from Homo sapiens (P04839) was generated using ProMod3 3.2.0, with 7d3e.1.A as the template model based on its FASTA sequence. The target sequence underwent a BLAST search against the primary amino acid sequence in the SMTL, resulting in the identification of 8 templates. An initial HHblits profile was constructed following the procedure outlined in [54], involving 1 iteration of HHblits against Uniclust30 [55]. The obtained profile was then compared to all profiles in the SMTL, revealing a total of 226 templates. Quality prediction for each identified template was based on features of the target-template alignment, with the highest-quality templates selected for model construction. Models were built using ProMod3, where coordinates conserved between the target and template were copied, and insertions/deletions were remodeled using a fragment library. Sidechains were reconstructed, and the model’s geometry was regularized using a force field. If loop modeling with ProMod3 was unsuccessful, an alternative model was created with PROMOD-II [56]. The overall and per-residue model quality was assessed using the QMEAN scoring function [57] and Ramachandran plot.

### 2.16. Molecular Docking

The molecular docking of NADPH oxidase 2 and the peptides 1–35 previously described by [53] was performed on HPEPDOCK webserver http://huanglab.phys.hust.edu.cn/hpepdock/ (accessed on 7 January 2021) [58] with a hierarchical algorithm for flexible and blind docking, followed by fast modeling of peptides’ conformation and sampling of binding orientations. The interaction networks between NADPH oxidase 2 and peptides 1–35 were determined using the Residue Interaction Network Generator (RING) webserver (http://protein.bio.unipd.it/ring, accessed on 7 January 2021), [59] with a strict distance threshold and closest network policy with multiple interaction types.

### 2.17. Statistical Analysis

The results are expressed as mean ± SEM. The normality of variables was assessed using the Kolmogorov–Smirnov test. A one-way analysis of variance (ANOVA) was conducted, followed by Tukey’s post hoc test. For the analysis of histological scores, the Kruskal–Wallis non-parametric test was employed, with Dunn’s post hoc test. Significance between groups was considered when p < 0.05. The analyses were executed using the Graphpad Prisma software (Prisma version 8.0, GraphPad Software, Inc., San Diego, CA, USA).

## 3. Results

### 3.1. Effect of Kefir on Gross Morphology in Indomethacin-Induced Gastric Ulcer in Mice 

**Figure 1:**
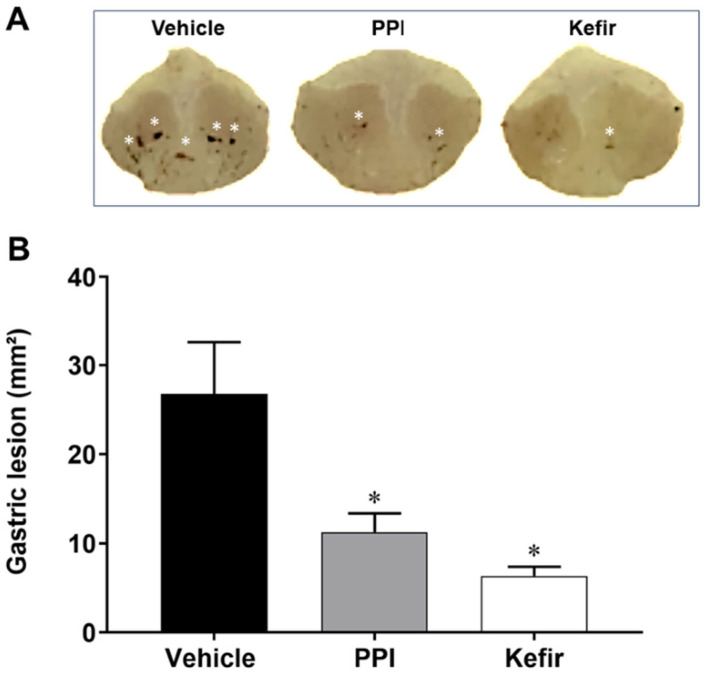
(**A**). Macroscopic photographs showing the stomachs of the experimental groups (Gastric lesions are represented by white asterisk). (**B**). Area of gastric lesion (mm^2^) in Vehicle (n = 6), PPI (n = 6), and Kefir (n = 6). The results are presented as the mean ± SEM. * *p* < 0.05 vs. Vehicle (ANOVA-1 one way, followed by Tukey post hoc).

Figure 1 shows typical stomach photographs from the experimental groups (Figure 1A) along with the quantification of the gastric lesion area (Figure 1B). Pretreatment with both PPI and kefir resulted in a similar reduction in gastric lesion size (12.40 ± 8.31 and 6.36 ± 3.22 mm^2^, respectively) compared to the Vehicle group (20.78 ± 17.22 mm^2^). 

### 3.2. Effects of Kefir on Ulcer and Protection Indexes 

Table 1 shows the parameters of gastric index (score 0–5), ulcer protection (%), and gastric juice pH. The Vehicle group had a higher gastric index score than the PPI and Kefir groups. Both PPI and kefir administration effectively prevented the development of gastric lesions, as determined by the percentage of ulcer protection. Interestingly, there were no significant differences in the pH values of gastric juice among the experimental groups.

### 3.3. Effects of Kefir on ROS Production in Gastric cells of Indomethacin-Induced Gastric Ulcer in Mice

**Figure 2:**
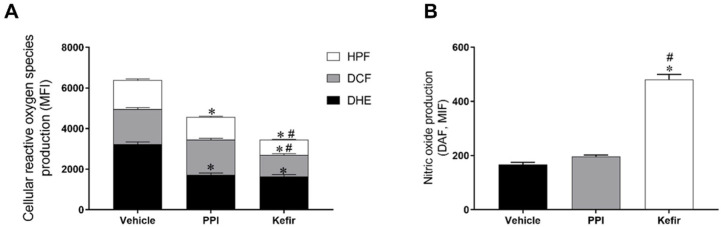
(**A**): Analysis of the production of ROS O_2_^−^, H_2_O_2_, and ONOO^−^/.OH^−^ in the gastric cells of the experimental groups. (**B**): Analysis of the bioavailability of NO in the gastric cells of the experimental groups. Results are expressed as the mean ± standard error of the mean. * *p* < 0.05 vs. Vehicle. # *p* < 0.05 vs. PPI (one-way ANOVA, followed by Tukey’s post hoc). Vehicle (n = 6), PPI (n = 6), and Kefir (n = 6).

Figure 2A shows the production of ROS in isolated gastric cells. The Vehicle group had an increased level of O_2_^−^ (3235 ± 105.9 a.u.) compared to the PPI (1707 ± 100.8 a.u.) and Kefir (1635 ± 93.45 a.u.) groups. Pretreatment with milk kefir (1068 ± 57.14 a.u.) decreased H_2_O_2_ production in gastric cells compared to the other groups (Vehicle: 1719 ± 76.79 and PPI: 1745 ± 64.07, a.u.). When evaluating the production of ONOO^−^/OH^−^, we observed that the animals that received vehicle (1433 ± 55.76 a.u.) had higher levels of this ROS, while the PPI (1123 ± 26.81 a.u.) and Kefir (744.3 ± 66.6 a.u.) groups showed lower levels. These results demonstrate the antioxidant action of milk kefir on gastric cells.

Figure 2B shows the nitric oxide (NO) bioavailability in gastric cells. It is evident that the NO bioavailability was increased in the animals that received kefir pretreatment (480.7 ± 18.97 a.u.) compared to the animals that received the vehicle (167.5 ± 7.843 a.u.) and PPI (197 ± 5.183 a.u.).

### 3.4. Effects of Kefir on Cell Apoptosis and DNA Fragmentation in Gastric Cells of Indomethacin-Induced Gastric Ulcer in Mice

**Figure 3:**
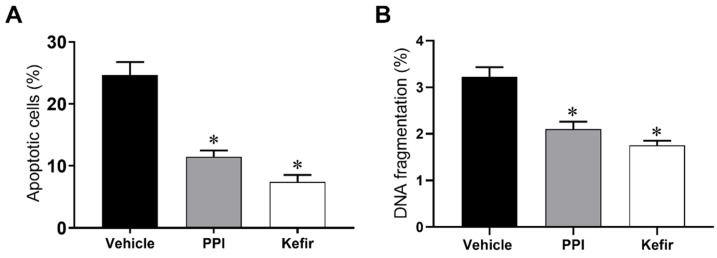
Evaluation of apoptosis (**A**) and cellular content evaluation (Sub G0 phases) (**B**) of gastric cells in the experimental groups. Results are expressed as the mean ± standard error of the mean. * *p* < 0.05 vs. Vehicle (one-way ANOVA, followed by Tukey post hoc). Vehicle (n = 6), PPI (n = 6), and Kefir (n = 6).

Figure 3A show the percentage of apoptosis of isolated gastric cells. The Vehicle group showed a significant increase in the percentage of apoptosis (24.6 ± 2.1%). In contrast, the PPI and Kefir groups showed a simultaneous decrease in apoptosis (PPI: 11.5 ± 1.0; Kefir: 7.4 ± 1.1%). 

DNA content was determined to assess the antigenotoxic effect of milk kefir (Figure 3B). The Vehicle group (3.2 ± 0.2 %) showed an increased percentage of cells in the subG0 phase when compared to the PPI (2.1 ± 0.1%) and Kefir groups (1.7 ± 0.1%).

### 3.5. Effects of Kefir on Systemic Cytokine Levels and Myeloperoxidase Activity in Plasma of Indomethacin-Induced Gastric Ulcer in Mice

Table 2 shows systemic levels of inflammatory cytokines and MPO activity. The pretreatments with Kefir (30.2 ± 2.7 pg/mL) significantly decreased IL-10 levels when compared to the other groups (Vehicle: 24.9 ± 3.2 and PPI: 23.5 ± 3.9, pg/mL). Furthermore, plasma levels of TNF-α in the PPI (38.8 ± 3.0 pg/mL) and Kefir (34.7 ± 4.9 pg/mL) groups were significantly lower than those in the Vehicle group (56.2 ± 5.5 pg/mL). Notably, vehicle, PPI, and kefir pretreatments did not significantly affect MPO activity (Vehicle: 0.042 ± 0.012; IBP: 0.050 ± 0.011; Kefir: 0.040 ± 0.010, au/mg protein).

### 3.6. Kefir Effects on Gastric Mucus and Histopathological Alterations in Gastric Tissue of Indomethacin-Induced Gastric Ulcer in Mice

**Figure 4:**
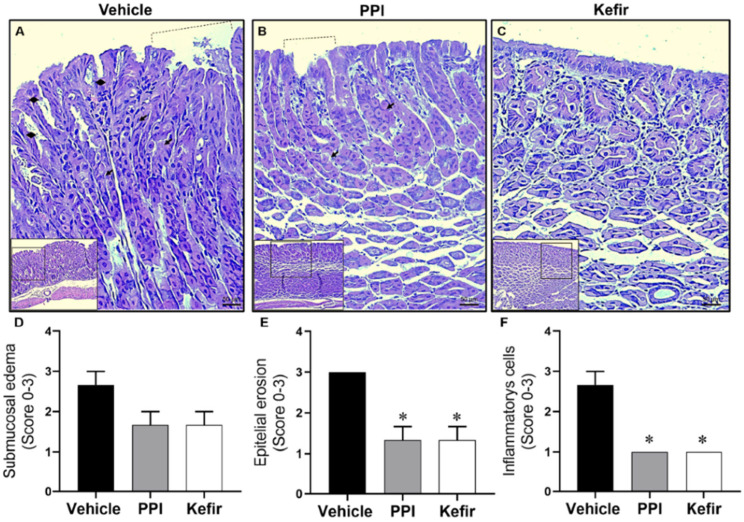
Histopathological images from gastric epithelium: Vehicle (**A**), PPI (**B**), and Kefir (**C**). Dashed lines represent gastric lesion, diamonds indicate the rupture of the epithelial layer, and the arrows denote the gastric glands. Images were captured using 10× and 20× objectives for enhanced clarity. Bar graphs present histological scores epithelial erosion (**D**), inflammatory cells (**E**), and submucosa edema (**F**). Data expressed as mean ± SEM. * *p* < 0.05 vs. Vehicle. The statistical analysis used the Kruskal-Wallis non-parametric test (ANOVA-1 via) followed by Dunn’s post hoc. Vehicle (n = 5), PPI (n = 5), and Kefir (n = 5).

Figure 4A–C shows typical photomicrographs of stomachs stained with HE at 20× and 40× magnification. The Vehicle group showed a gastric ulcer with epithelial erosion (Figure 4A), while the PPI and kefir pretreatment effectively prevented the injury (Figure 4B,C), demonstrating their potential as therapeutic interventions. In addition, the kefir pretreatment showed preservation of the glandular structure in the gastric tissue (Figure 4C), highlighting its beneficial effects on tissue integrity.

Figure 4D–F show the scores obtained from histopathological analysis of gastric tissue. The evaluation of submucosa edema in the stomach did not show a significant difference between the experimental groups (Figure 4D). However, kefir pretreatment (1.33 ± 0.57; 1.00 ± 00) and PPI pretreatment (1.33 ± 0.57; 1.00 ± 00) showed a reduction in the epithelial erosion (Figure 4E) and inflammatory cells (Figure 4F) compared to the Vehicle group (3.00 ± 00; 2.66 ± 0.57).

**Figure 5:**
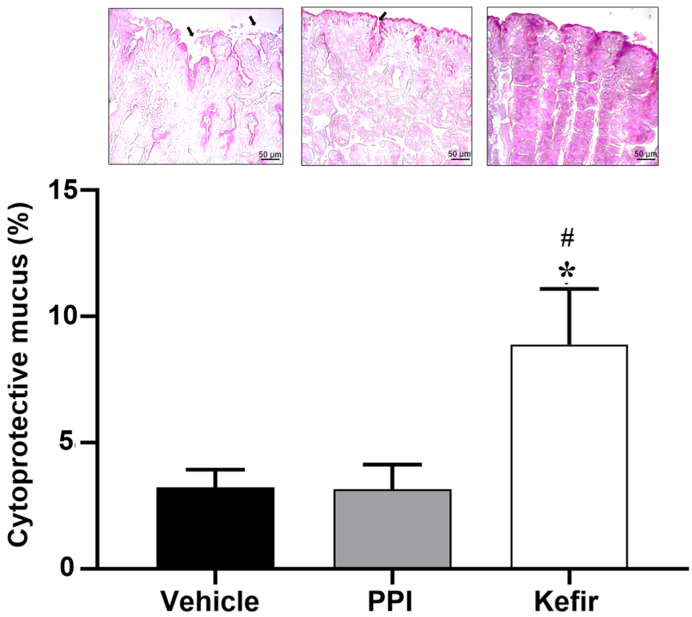
(**Top panel**) shows histopathological images of the gastric epithelium stained with PAS to evaluate a production of cytoprotective mucus from the experimental groups Vehicle, PPI, and Kefir. The bar graph evaluated the % of cytoprotective mucus. Gastric lesions are represented by black arrows and mucus production is represented by the intensity of the stain. Images obtained by 20× objectives. * *p* < 0.05 vs. Vehicle. # *p* < 0.05 vs. PPI (one-way ANOVA, followed by Tukey post hoc). Vehicle (n = 5), PPI (n = 5), and Kefir (n = 5).

Figure 5 (top panel) shows photomicrographs of the cytoprotective mucus present in the gastric tissue of the experimental groups. Figure 5 graph shows the percentage of cytoprotective mucus in the experimental groups. The Vehicle group (3.21 ± 0.71%) showed a reduction in the cytoprotective mucus in the gastric tissue. The PPI group (3.152 ± 0.97%) showed preservation of the mucus layer despite the presence of some ulcerative lesions, compared to the Vehicle group. The administration of milk kefir (8.89 ± 2.19%) preserved the cytoprotective layer of mucus more than the other groups.

**Figure 6:**
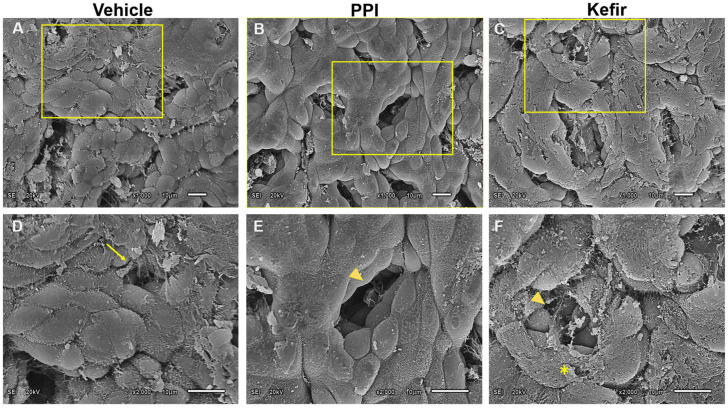
Scanning electron photomicrographs show beneficial effects of kefir administration on gastric epithelium. Detailed images revealed the remarkable differences among the groups (Vehicle, PPI, and Kefir). (**A**): Vehicle, ×1000; (**B**): PPI, ×1000; (**C**): Kefir, ×1000; (**D**): Vehicle, ×2000; (**E**): PPI, ×2000, and (**F**): Kefir, ×2000. Arrow: gastric lesion; arrow head: gastric glands; asterisk: extra layer of mucosal epithelial tissue. Vehicle (n = 2), PPI (n = 2), and Kefir (n = 2). The arrow indicates a gastric lesion, the arrowhead points to gastric lands and the asterisk denotes an extra layer of mucosal epithelial tissue.

The Vehicle group (Figure 6A,D) shows a disorganized gastric epithelium with cell erosion, leading to cell loss and epithelial damage. The PPI group (Figure 6B,E) shows a slight desquamation of the gastric epithelium with mild epithelial damage, but the gastric pits are preserved. The Kefir group (Figure 6C,F) exhibits intact epithelial tissue with regular arrangement of epithelial cells without erosions. The gastric pits are preserved and slightly enlarged, indicating the proliferation of epithelial cells in the Kefir group.

### 3.7. Bioprospection of Antioxidant Peptides from Kefir and the Favorable Interaction with NADPH Oxidase 2

As described before, the peptides selected for this analysis were derived from a proteomic investigation of kefir conducted by [53]. Subsequently, these peptides underwent docking experiments with NADPH oxidase 2 to explore their potential antioxidant activities. Reference [53] characterized the physicochemical features of these peptides, and in silico analyses revealed their non-toxic nature. NADPH oxidase was selected for its robust association with ROS production in gastric cells, given the established correlation between gastric oxidative stress and the upregulation of NADPH oxidase [60,61,62,63]. It is crucial to emphasize that this represents one of the possible mechanisms for investigation. Subsequent stages involve evaluating the potential in vitro and in vivo antioxidant properties of peptide 13, as well as elucidating its gastroprotective effects. 

**Figure 7:**
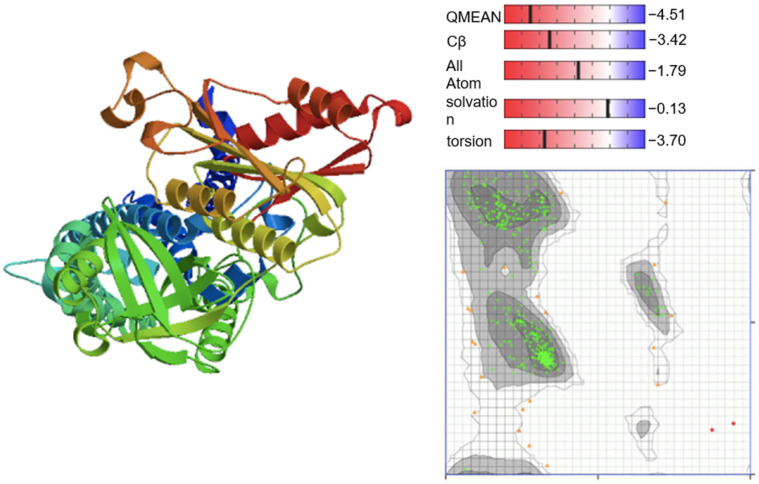
PDB structure of Homo sapiens NADPH oxidase 2 (P04839). The PDB structure was validated with QMEAN scoring function and Ramachandran Plot. The Ramachandran Plot visualizes the energetically allowed regions of NADPH oxidase 2 at different angles ψ against φ of amino acid residues. The green clusters represent the most favored region (489 residues = 95.508%) while the grey region indicates the generously allowed region (21 residues = 4.102%). The red dots are disallowed regions (2 residues, 0.391%).

Figure 7 shows the PDB structure of Homo sapiens NADPH oxidase 2 (accessed on 7 January 2021).

A total of 35 peptides were docked with NADPH oxidase 2 and further clustered into the 10 most significant models according to the peptide conformation. The cluster with the lowest dock scoring was filtered and tabulated in Table 3. The dock scoring arbitrary reflects the relative ranking of peptide binding affinity. As illustrated by Table 3, peptide 13 (IPPLTQTPVVVPPFLQPEVMGVSK) exhibited the lowest docking score of −278.492 kcal/mol, suggesting its first ranking as the peptide, which displayed relatively higher binding affinity.

A set of predicted five interface residues were determined by InterEvDock (accessed on 7 January 2021) after physics-based potential (FRODOCK scores), statistically optimized atomic potential (SOAP-PP), and InterEV score. These interface residues are those residues most likely involved in the interface, predicted from the best models. The top five residues were listed in Table 4 and used for information-driven flexible docking on HADDOCK 2.2 (easy interface).

**Figure 8:**
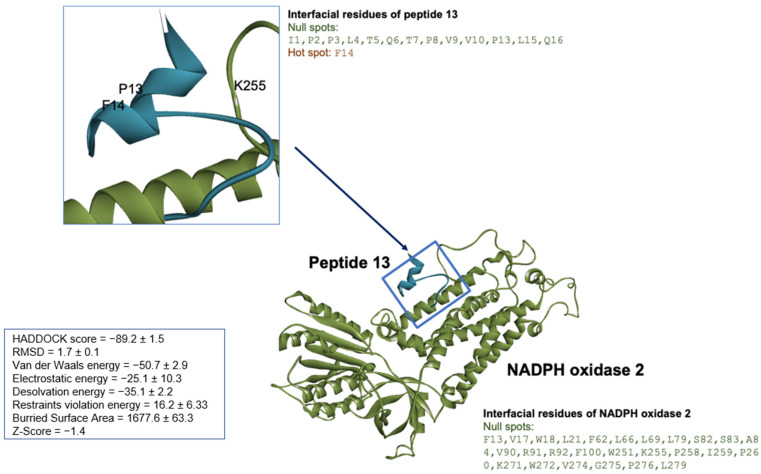
The peptide 13/NADPH oxidase 2 molecular docking using HPEPDOCK and HADDOCK. The docked structure was illustrated by Discovery Studio Visualizer version v19.1.0.18287 (BIOVIA, San Diego, CA, USA). The NADPH oxidase 2 represented in green solid ribbon cartoon and peptide 13 was colored in cyan blue.

The binding orientation of peptide 13 and NADPH oxidase 2 was attained by defining the interface residues for each biomolecule as active residues, while the passive residues were automatically defined around active residues. The easy interface binding orientation revealed the binding sites of peptide 13 to NADPH oxidase 2 (Figure 8). A total of 134 docked structures were clustered into 12 clusters according to HADDOCK scores. The HADDOCK scores ranged from −89.2 ± 1.5 (the best cluster 1) to −46.1 ± 6.3 (the worst cluster 9). Thus, the top-ranked peptide 13 cluster—cluster 1—was then subjected to RING 2.0 analysis to determine the docking pose and interaction forces. Based on RING analysis on cluster 1, the main interaction forces within the peptide 13/NADPH oxidase 2 were Van der Waals (48.29%) and hydrogen bonding (48.01%). The ionic bonding and π-π stacking constituted about 0.83% and 2.87%, respectively. The interfacial residues on peptide 13/NADPH oxidase 2 were identified and classified as hotspots and null spots residues by SpotOn webserver (accessed on 7 January 2021). The residues involved in the interaction were (a) peptide 13: I1, P2, P3, L4, T5, Q6, T7, P8, V9, V10, P13, L15, Q16 being null spot residues and F14 as hotspot residues; (b) NADPH oxidase 2: F13, V17, W18, L21, F62, L66, L69, L79, S82, S83, A84, V90, R91, R92, F100, W251, K255, P258, 259, P260, K271, W272, V274, G275, P276, L279, which revealed possible binding sites distinguishable from the iron and flavin nucleotide-binding sites on the enzyme.

## 4. Discussion

In recent years, there has been an increase in scientific research on kefir, stimulated by its association with potential therapeutic benefits, particularly its antioxidant potentials [14,41,42]. In this current investigation, we have elucidated the capacity of kefir to prevent the development of gastric ulcers in an experimental model of gastric injury induced by indomethacin. Our findings attributed the gastroprotective effect of kefir to its potent antioxidant properties. Specifically, we showed a significant reduction in intracellular ROS accumulation, concomitant with an elevation in NO levels, culminating in a marked attenuation of DNA damage and apoptosis, which reinforces gastric tissue defense mechanisms. Moreover, we demonstrated that peptides within kefir intricately interact with NADPH oxidase 2. Our study demonstrated that kefir administration yields systemic effects by orchestrating the regulation of inflammatory cytokines, as manifested by a reduction in TNF-α levels and a concomitant augmentation of IL-10, thereby highlighting the anti-inflammatory potential of this probiotic.

In the present study, we induced gastric ulcers in mice through the administration of indomethacin. It is noteworthy that chronic use of NSAIDs has been linked to gastrointestinal toxicity [3]. This toxicity is primarily attributed to the disruption of the gastric mucosal epithelium and the systemic reduction in prostaglandins, which are predominantly derived from COX-1 and are pivotal for maintaining the structural integrity of the gastric tissue [2]. 

In recent years, probiotics have emerged as a promising therapeutic avenue for the prevention of various diseases, particularly those affecting the gastrointestinal tract [14,64,65]. Our study showed that kefir is as effective as the reference treatment (PPI) in preventing gastric lesions, aligning with the findings of previous research [14,18]. The gastroprotective attributes of milk kefir are due to its probiotic properties, particularly those associated with lactobacilli and bifidobacteria [66,67]. Lactobacilli is renowned for its ability to extend healing time and maintain the integrity of the mucosal barrier [67,68,69]. In the present study we maintain a consistent kefir dosage of 0.3 mL of kefir per 100 g of the animal’s weight. This decision is grounded in our extensive research experience, as evidenced by our previous publication [14,24,31,53,70,71]. Positive outcomes in reducing gastric lesions were demonstrated by [14] using this specific dosage. Furthermore, we align our approach with the principles of the 3Rs (reducing, replacement, and refinement), as advocated by the ethical committee for animal use, to minimize the impact on experimental animals. However, given the observed effects at this predetermined dose, future assays employing varying concentrations may be useful to elucidate whether the effects of kefir exhibit a dose-dependent relationship, discerning whether the protection would be mediated by the microbiota per se and/or by metabolites of kefir. 

Considering that metabolites from microorganisms may contribute to health properties of the fermented products, it should be taken into account that kefir bioactive compounds can prevent gastric lesions by interrupting oxidative stress and modulating immune system responses [32,72]. Recently, ref. [73] showed that the HPLC chromatogram analysis of kefir polyphenolic compounds revealed the existence of gallic acid and other organic compounds. Gallic acid has demonstrated robust antioxidant properties [74,75] and has been found to safeguard the gastric mucosa from ethanol, ischemia-reperfusion, NSAID, and aspirin-induced gastric lesions in rats [76,77,78,79]. As a result, we suggest that gallic acid may play a significant role as one of the contributors to the gastroprotective effect of kefir. In addition, gallic acid exhibits a potent immunomodulatory effect through the inhibition of ROS/NF-kB/TNF-α signaling pathway [80]. Additionally, it mitigates gastric mucosal lesions by decreasing acid output, elevating antioxidant levels (SOD, catalase, and glutathione peroxidase), reducing myeloperoxidase, and lipid peroxidation, suppressing the release on TNF-α, and promoting the endogenous defensive factor prostaglandin E2 [76,77,78,79].

Prior investigations established that the etiology of gastric mucosal damage predominantly is due to the injurious repercussion of oxidative stress. Kefir metabolites can prevent gastric lesions by interrupting oxidative stress and modulating immune system responses [32,72]. Polysaccharides, peptides, phenolic compounds, and short chain fat acids (SCFA) are well known kefir metabolites [72,73,81]. The experimental model of gastric ulcer induced by NSAIDs directly correlates with oxidative stress, caused by the increased production of ROS [13,14,82,83]. In the present study, we have demonstrated that the administration of milk kefir significantly reduces the cytoplasmic production of ROS within isolated gastric cells. Similar effects were demonstrated by [14,18], who reported analogous reductions in local and systemic oxidative stress following kefir administration, providing further evidence of its gastroprotective effects in experimental models of gastric injury. These antioxidant effects are postulated to be linked to increased activity of crucial antioxidant enzymes (superoxide dismutase, catalase, and glutathione peroxidase) [84] and decreased oxidative damage to macromolecules [14,85,86].

Oxidative stress plays a pivotal role in the initiation of gastric lesions. Reference [14] previously delineated the bidirectional relationship between the systemic antioxidant effects of kefir and gastroprotection, serving as inspiration for our current work. We conducted a bioprospection to identify potential antioxidant peptides and assessed the local actions of kefir in gastric lesions. It is important to note that the pathophysiology of gastric ulcer involves the upregulation of NADPH oxidase, which results in the heightened production of ROS [62,87]. In our study, we have substantiated that 35 peptides derived from milk kefir engage in specific interactions with NADPH oxidase 2 (NOX2). This interaction was prompted by a preceding publication from our group, which had initially unveiled the bioactive potential harbored within small peptides sourced from kefir [53]. Importantly, we observed that all 35 peptides exhibited docking scores of at least −140 kcal/mol, with peptide 13 having the highest docking score (−278.492 kcal/mol assessed through molecular docking with NOX2). Reference [32] demonstrated the antioxidant power of a kefir-derived peptide named KEF-1, achieved through the attenuation of the NADPH oxidase-mediated pathway’s contribution. These findings are primarily due to the presence of bioactive compounds generated through the metabolic activities of microorganisms during the fermentation and storage process of the beverage [53]. Consequently, the antioxidative impact attributed to milk kefir presumably arises from the existence of antioxidant peptides, which manifest the remarkable capability to reduce both ROS generation and lipid peroxidation [32,88]. 

Peptides have gained significant attention as bioactive fragments present in the sequences of food proteins. Fermented dairy products, like kefir, serve as reservoirs for bioactive peptides derived from native proteins, exhibiting measurable physiological effects and positive impacts on human health [41,42,53]. In a previous study, we unveiled the abundance of biofunctional peptides in cow milk [53]. The action of microbial proteases during fermentation substantially enhances the release of peptides from all proteins. Antioxidant effects in kefir from sheep and cow milks have been demonstrated [32,88,89]. Reference [88] demonstrated antioxidant activity in fourdifferent peptides with the final amino acid sequence VRGPFP, while [89] suggested antioxidant effect of the VYPFPGPIPN peptide, and [32] showed antioxidant effect in vivo of the peptide AVPYPQR.

Nitric oxide (NO) is a very small biologically potent molecule. Studies have shown that NO plays a defensive role in mitigating gastric erosion and ulceration [14,90]. In light of these findings, NO modulates the recuperative processes associated with gastric ulcers. This modulation includes the augmentation of blood circulation, the facilitation of new blood vessel formation within the ulcerated region, and the production of cytoprotective mucus [67,91]. Undoubtedly, operating as a vasodilator, NO maintains a consistent blood supply to the gastric mucosa. Our data suggest that the NO pathway might constitute one of the underlying mechanisms by which the probiotic kefir exerts its gastroprotective effects.

The process of gastric ulcer healing entails a carefully orchestrated interplay of various mechanisms, restoring the equilibrium between deleterious and protective factors [13,14,92,93]. Among the probiotics extensively investigated for treating and preventing gastrointestinal disorders, lactobacilli and bifidobacteria species emerge as leading candidates [24,69,94]. These bacterial species exhibit remarkable resilience in the face of the challenging luminal conditions within the gastrointestinal tract [95,96]. Furthermore, previous studies have demonstrated the efficacy of lactobacilli and bifidobacteria in facilitating the recovery of gastric ulcers [69,97,98,99]. Probiotics have a dual capacity, to inhibit the formation and accelerate the healing of gastric ulcers. These beneficial actions of probiotics can be attributed to a variety cellular and molecular mechanisms [14,99]. 

Probiotics also play a pivotal role in preserving the integrity of the gastric mucosal barrier through various mechanisms, including the augmentation of mucus secretion, promotion of cell proliferation, and the inhibition of apoptosis [14,99]. Reference [100] has provided evidence that probiotics can strengthen the gastric mucosal barrier by inhibiting apoptosis. The continual renewal of cells constitutes a crucial factor in maintaining the integrity of the gastric mucosal barrier. In the present study, we demonstrated that the use of milk kefir prevents DNA damage and apoptosis in gastric cells. Previous studies have demonstrated that pretreatment with *Lacticaseibacillus rhamnosus* results in a significant reduction in the number of apoptotic cells within ethanol-induced gastric ulcers [69]. Additionally, *Lacticaseibacillus rhamnosus* stimulates the proliferation of these cells [101], thereby fostering the regeneration of epithelial cells, particularly on the regions adjacent to the ulcers. Furthermore, we have previously showed that kefir administration reduced DNA damage and apoptosis systemically [14].

Probiotics play pivotal roles in both the prevention and treatment of gastric ulcers induced by NSAIDs [10]. The augmentation of innate and adaptative immune responses, along with their correlated anti-pathogenic and anti-inflammatory activities are well-documented as beneficial effects of probiotics [102]. According to [103], kefiran produced by *Lactobacilus kefiranofaciens* has the ability to modulate the gut mucosal immunological response. Furthermore, [104] demonstrated in vitro antioxidant and anti-inflammatory properties of kefiran, highlighting its effectiveness as a scavenger for ROS, suggesting its potential as an important candidate for promoting tissue repair and regeneration. Investigations focusing on gastric ulcers have consistently shown that probiotic administration leads to a reduction in gastric mucosal damage scores, lipid peroxidation, and levels of pro-inflammatory cytokine, like TNF-α [97,99,105]. Within this context, in the current study, we have observed that pretreatment with milk kefir causes a significant reduction in plasma TNF-α levels while concomitantly elevating IL-10 levels. Notably, TNF-α assumes a crucial role as a pro-inflammatory cytokine in the context of gastric injury. It actively participates in the recruitment of leukocytes, thereby amplifying local inflammation and subsequently elevating levels of ROS, resulting in epithelial damage, and triggering a pro-apoptotic response [20,83,106]. Conversely, IL-10, a prominent anti-inflammatory cytokine, plays a pivotal role in counteracting the effects of pro-inflammatory cytokines, particularly TNF-α [107]. These findings offer further support for the gastroprotective effects of milk kefir, attributed to its potent anti-inflammatory properties, likely stemming from the presence of bioactive molecules in the complex mixture of kefir. It is well-established that fermented milk products serve as natural sources of bioactive peptides, generated during the hydrolysis of milk proteins by microbial proteases within the starter culture. Therefore, a comprehensive understanding contributes to an enhanced grasp of the potential therapeutic applications of milk kefir in preventing NSAID-induced gastric ulcers and promoting gastrointestinal health.

The process of gastric ulcer healing involves a coordinated response interplay of various mechanisms that collaborate to rectify the imbalance between deleterious and protective factors within stomach. Pretreatment of mice with probiotics alleviate histopathological changes, particularly the infiltration of inflammatory cells and gastric mucosal damage [97]. In animal models, the administration of *Bifidobacterium bifidum* has demonstrated a reduction in the degradation of the mucus barrier [98] and stimulated an increased production of gastric mucous [108]. The reduction in prostaglandin biosynthesis (through COX-1 inhibition by indomethacin) is directly linked to a decrease in mucus secretion, a factor closely tied to the pathophysiology of gastrointestinal mucosal diseases [109]. On the other hand, it is well described that SCFAs are able to increase mucus production and secretion [110]. Moreover, kefir microorganisms could increase the prostaglandin production, contributing to mucoprotection [111,112,113]. In human studies, [68] found that pretreatment with lactobacilli effectively shielded against the disruption of the gastric mucosal barrier induced by indomethacin. In our study, we observed a notable accumulation of inflammatory cells in the gastric tissue of the Vehicle group, while the presence of inflammation was significantly less pronounced in the PPI and Kefir groups. Furthermore, the Kefir group exhibited the preservation of gastric gland functionality, characterized by the presence of intact gastric glands. In contrast, the prolonged use of PPIs led to hyperplasia and hypertrophy of parietal cells, as corroborated by [114,115]. Interestingly, mice that received pretreatment with kefir displayed an additional lining layer in the stomach epithelium, potentially linked to interactions with mucin or other gastric structures, as reported by [116].

In our investigation, we evaluated the protective potential of kefir when administered prior to ulcer induction, mirroring prophylactic clinical procedures. The results revealed that the therapeutic efficacy of kefir can be attributed to its antioxidant properties, which are closely linked to the presence of diverse bioactive peptides within kefir. Furthermore, the beneficial impact of kefir was concurrent with its anti-inflammatory effects.

## 5. Conclusions

This study demonstrates that kefir possesses the potential to mitigate gastric mucosal damage induced by NSAIDs. The effects of kefir are attributed to its ability to enhance antioxidant and cytoprotective defenses, while simultaneously reducing oxidative stress and inflammation. These findings highlight the therapeutic potential of kefir in the context of NSAID-induced gastric ulcers. Furthermore, they open avenues for future clinical investigations, suggesting the inclusion of milk kefir as a gastroprotective adjuvant agent for managing NSAID-induced gastric ulcers. To advance the field, further research could explore specific mechanisms underlying kefir’s protective effects, including the peptide 13, and evaluate its efficacy in experimental and clinical studies. Despite the promising outcomes, it is essential to acknowledge the limitations of the study, such as the need of investigations using different kefir cultures with variations in microbiological and bioactive compounds composition. Finally, the findings of the study pave the way for future clinical investigations, fostering the incorporation of milk kefir as a gastroprotective agent for the management of NSAID-induced gastric ulcers.

## Figures and Tables

**Table 1:** Gastric index, ulcer protection (%), and gastric juice (pH) parameters in experimental groups.

Groups	Gastric Index	Ulcer Protection (%)	Gastric Juice (pH)
Vehicle	4.0 ± 0.3	-	4.91 ± 0.42
PPI	3.0 ± 0.2	64.33 ± 6.31 *	4.35 ± 0.37
Kefir	3.0 ± 0.3	69.39 ± 4.90 *	4.55 ± 0.25

* *p* < 0.05 vs. Vehicle (one-way ANOVA, followed by Tukey post hoc), n = 6 in each group.

**Table 2:** Levels systemic of IL-10, TNF-α, and MPO activity.

Groups	IL-10 (pg/mL)	TNF-α (pg/mL)	MPO Activity (a.u. mg Protein)
Vehicle	24.9 ± 2.0	56.2 ± 5.5 pg/mL	0.042 ± 0.012
PPI	23.5 ± 2.4 *	38.8 ± 3.0 pg/mL *	0.050 ± 0.011
Kefir	33.8 ± 2.6 *^#^	34.7 ± 4.9 pg/mL *	0.040 ± 0.010

* *p* < 0.05 vs. Vehicle. ^#^ *p* < 0.05 vs. PPI (one-way ANOVA, followed by Tukey post hoc). Vehicle (n = 8), PPI (n = 8), and Kefir (n = 8).

**Table 3:** The dock scoring of peptides 1–35 obtained from molecular docking with NADPH oxidase 2 (*Homo sapiens*, P04839).

#	Peptide Sequence	Dock Scoring (kcal/mol)
1	AVPYPQR	−179.717
2	CGLVPVLAENR	−211.662
3	DFGHIQYVAAYR	−236.526
4	DMPIQAFLLY	−202.306
5	EINQFYQK	−195.413
6	EMPFPKYPVEPF	−208.377
7	FLLYQEPVLGPVRGPFPIIV	−261.937
8	FVAPFPEVFG	−215.28
9	GLFQINNK	−165.179
10	GTWYSLAMAASDISLLDAQSAPLR	−215.777
11	IHPFAQTQSLVYPFPGP	−236.735
12	IHPFAQTQSLVYPFPGPIPNSLPQN	−245.583
13	IPPLTQTPVVVPPFLQPEVMGVSK	−278.492
14	KLDQWLCEK	−182.732
15	KVGINYWLAHK	−221.75
16	LACQCLVR	−172.099
17	LLDDDLTDDIMCVK	−141.894
18	LLYQEPVLGPVRGPFPIIV	−242.36
19	LVYPFPGPIHNSLPQ	−231.785
20	LVYPFPGPIPN	−212.428
21	LYQEPVLGPVRGPFPII	−217.374
22	LYQEPVLGPVRGPFPIIV	−220.833
23	NLHLPLPLLQ	−228.763
24	NLHLPLPLLQS	−194.289
25	PFPEVFGK	−210.473
26	QFLPYPYYAKPA	−233.97
27	RFFVAPFPEVFGK	−252.026
28	SLVYPFPGPIH	−228.771
29	SLVYPFPGPIHNSLPQ	−241.852
30	SQSLTLTDVENLHLPLP	−196.224
31	TDLLNVCMDAK	−166.611
32	VAPFPEVFGK	−195.564
33	WCTISQPEWFK	−217.273
34	YPFPGPIPN	−223.244
35	YPVEPFTESQSL	−189.741

**Table 4:** Top five residues in peptide 13 and NADPH oxidase 2 predicted to be involved interaction by InterEvDock.

Rank	NADPH Oxidase 2	Peptide 13
1	W18	I1
2	L21	F14
3	W272	P2
4	F62	P12
5	L66	V10

## Data Availability

The datasets used and analyzed in this paper are available from the corresponding author upon reasonable request.
